# StrainSeeker: fast identification of bacterial strains from raw sequencing reads using user-provided guide trees

**DOI:** 10.7717/peerj.3353

**Published:** 2017-05-18

**Authors:** Märt Roosaare, Mihkel Vaher, Lauris Kaplinski, Märt Möls, Reidar Andreson, Maarja Lepamets, Triinu Kõressaar, Paul Naaber, Siiri Kõljalg, Maido Remm

**Affiliations:** 1 Department of Bioinformatics, University of Tartu, Tartu, Estonia; 2 Institute of Mathematical Statistics, University of Tartu, Tartu, Estonia; 3 Synlab Eesti, Tallinn, Estonia; 4 Department of Microbiology, Institute of Biomedicine and Translational Medicine, University of Tartu, Tartu, Estonia; 5 United Laboratories, Tartu University Clinics, Tartu, Estonia

**Keywords:** *k*-mer, Clade, Strain identification, Species identification, Diagnostics

## Abstract

**Background:**

Fast, accurate and high-throughput identification of bacterial isolates is in great demand. The present work was conducted to investigate the possibility of identifying isolates from unassembled next-generation sequencing reads using custom-made guide trees.

**Results:**

A tool named StrainSeeker was developed that constructs a list of specific *k*-mers for each node of any given Newick-format tree and enables the identification of bacterial isolates in 1–2 min. It uses a novel algorithm, which analyses the observed and expected fractions of node-specific *k*-mers to test the presence of each node in the sample. This allows StrainSeeker to determine where the isolate branches off the guide tree and assign it to a clade whereas other tools assign each read to a reference genome. Using a dataset of 100 *Escherichia coli* isolates, we demonstrate that StrainSeeker can predict the clades of *E. coli* with 92% accuracy and correct tree branch assignment with 98% accuracy. Twenty-five thousand Illumina HiSeq reads are sufficient for identification of the strain.

**Conclusion:**

StrainSeeker is a software program that identifies bacterial isolates by assigning them to nodes or leaves of a custom-made guide tree. StrainSeeker’s web interface and pre-computed guide trees are available at http://bioinfo.ut.ee/strainseeker. Source code is stored at GitHub: https://github.com/bioinfo-ut/StrainSeeker.

## Introduction

Pathogenic bacteria represent a considerable danger for human health worldwide. For effective outbreak detection and epidemiological surveillance, bacterial pathogens must be rapidly identified. For this, the pathogen is usually isolated and various molecular typing methods used, most are based on polymerase chain reaction, or, in the last few years, whole-genome sequencing (WGS) (Inouye et al., 2014; Hasman et al., 2014). Matrix-assisted laser desorption/ionization time-of-flight mass spectrometry has also been used to quickly and cheaply identify bacterial colonies (Karamonová et al., 2013), but for strain-level identification it requires very precise, manually crafted databases for each species which, to a large extent, are not available today.

One of the main goals of molecular typing is classification of pathogens into clonal groups (Inouye et al., 2014). This is important because strains from the same species can have vastly different effects on their host. A well-known example is *Escherichia coli*, a species which contains some strains such as *E. coli* O157:H7 (Tu, He & Zhou, 2014) and *E. coli* EC958 (Petty et al., 2014) that are considerably more virulent than others. For classifying isolates, multi-locus sequence typing (MLST) (Maiden, 2006) or clone-specific markers have been used (Inouye et al., 2014). Several approaches have been developed that can detect clinically relevant mutations and alleles directly from WGS reads, such as KvarQ (Steiner et al., 2014), Mykrobe (Bradley et al., 2015) and SRST2 (Inouye et al., 2014). However, in most cases deep sequencing coverage and highly specialized allele databases are required (e.g., Mykrobe can be used only for *Mycobacterium tuberculosis* and *Staphylococcus aureus* identification), making the use of such programs complicated for the identification of isolates. The Reads2Type web service (Saputra et al., 2015) can be used for the rapid taxonomical identification of any bacterial isolate, but only at the species level. To classify an isolate to a clonal group, higher resolution is necessary.

Instead of looking for a set of clone-specific markers, full bacterial genomes could be used as the reference sequences. Bacterial identification programs based on the detection of short DNA oligomers with length *k* (*k*-mers) such as Kraken (Wood & Salzberg, 2014) or CLARK (Ounit et al., 2015) can use the whole RefSeq bacterial genomes database and identify isolates with high accuracy (Saputra et al., 2015). Moreover, they can handle low-coverage WGS samples as well, because they classify each read separately. Compared to the alignment-based tools like Sigma (Ahn, Chai & Pan, 2015), *k*-mer based programs have shown to be superior, especially when considering running time (Lindgreen, Adair & Gardner, 2016; Peabody et al., 2015). Kraken identifies each of the sequence reads separately using the National Center for Biotechnology Information (NCBI) taxonomy tree, counting the hits to each of the taxons on the tree and finding the branch with the most total hits. CLARK also identifies each of the reads, but instead of using a tree, it is based on a non-hierarchical, user-defined database.

We present StrainSeeker, a program for quick classification of bacterial isolates into clonal groups or clades direct from raw WGS sequencing reads. StrainSeeker uses a guide tree to approximate phylogenetic relationships between reference bacterial genomes down to the strain level, not being tied to existing taxonomic systems such as the NCBI taxonomy. This helps to avoid controversies such as the case of *E. coli* and *Shigella* sp., by which *Shigella* strains have been shown to be phylogenetically very similar to *E. coli*, but belong to different species according to NCBI taxonomy (Lan & Reeves, 2002). The guide tree has to be provided by the user. We developed a novel algorithm that assigns the isolate to a specific clade on the guide tree, based on the number of shared *k*-mers on different taxonomic levels. Instead of read counts assigned to individual reference genomes, StrainSeeker results are given as a single strain or a clade consisting of multiple strains, along with a visual representation of the guide tree showing where the isolate branches off.

### Implementation

**Figure 1:**
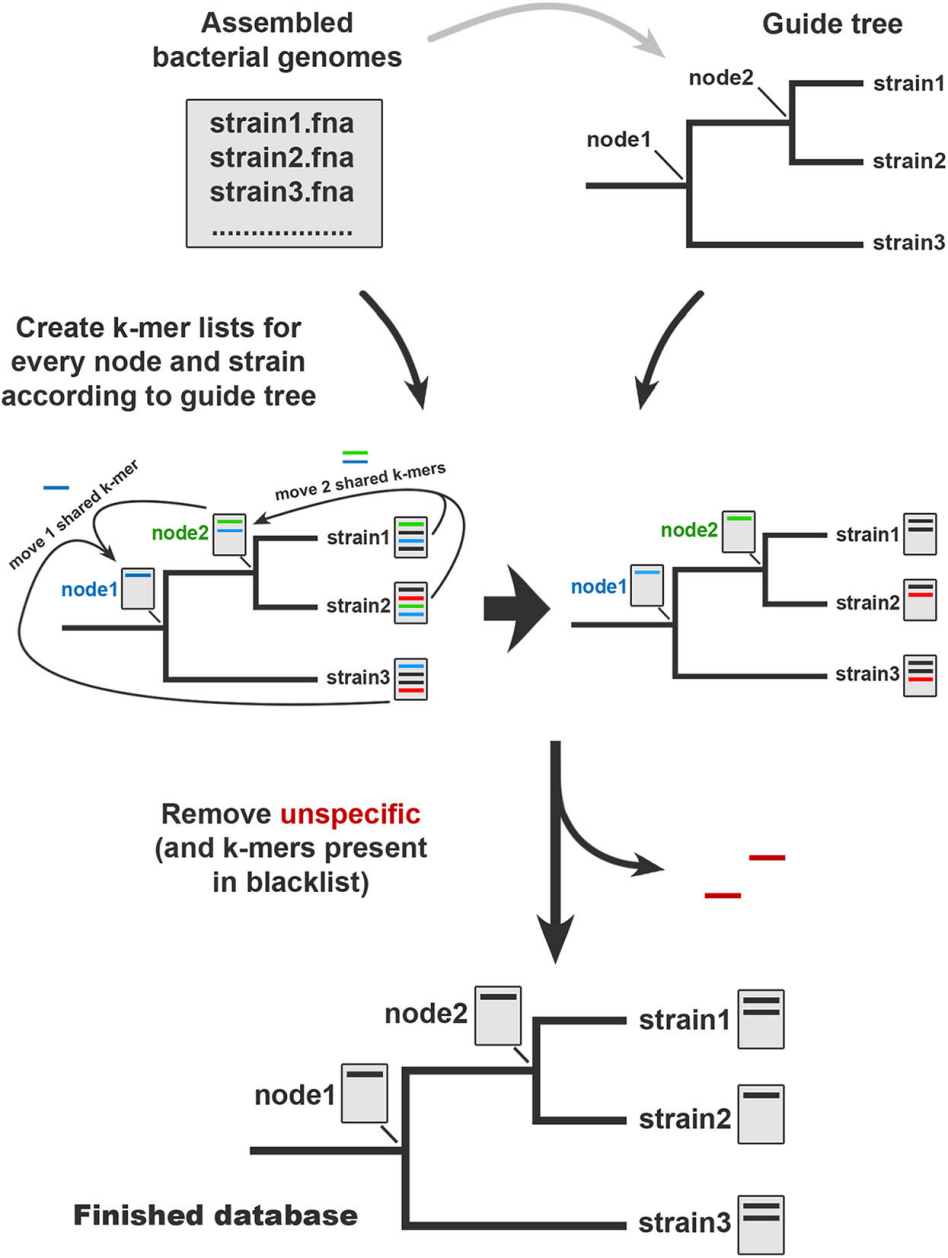
StrainSeeker database building process. Database construction requires high-quality assembled bacterial genomes as input. Next, the user has to build a guide tree that contains all the input strains. Before the building process, the assembled genome of each strain is converted into a *k*-mer list. Building process starts from the strain level and moves shared *k*-mers toward the root. The final step is to eliminate non-specific *k*-mers that occur in the “blacklist” or in any other nodes. The finished database contains *k*-mer lists specific to each node and strain and can be used to quickly identify any strain included on the guide tree and the strains related to them.

StrainSeeker is designed to analyze raw WGS sequencing reads and quickly determine the clade of the isolate in the user-provided guide tree. Before StrainSeeker can be used to identify bacteria, the database of specific *k*-mers needs to be built or a pre-built one downloaded. To create a database, the user needs to provide a set of high-quality assembled bacterial strain genomes and a guide tree describing the approximate phylogeny of provided strains (Fig. 1). Any Newick-format tree can be used as the guide tree. The database is built according to the guide tree structure, starting from the leaves (individual strains) and moving toward the root. All operations with *k*-mers are done using the GenomeTester4 software (Kaplinski, Lepamets & Remm, 2015). To reduce the noise in samples that may be caused by the DNA of other, non-bacterial organisms such as human DNA in clinical samples, the user can also provide a list of potential contaminating sequences (the “blacklist”). In the database building process, all strain *k*-mers that are present in the blacklist are eliminated. The blacklist itself is not part of the database. The final database contains specific *k*-mers for each node and leaf (strain) represented in the guide tree and an index file containing the database structure and *k*-mer counts. The database has to be built only once, not for every identification.

**Figure 2:**
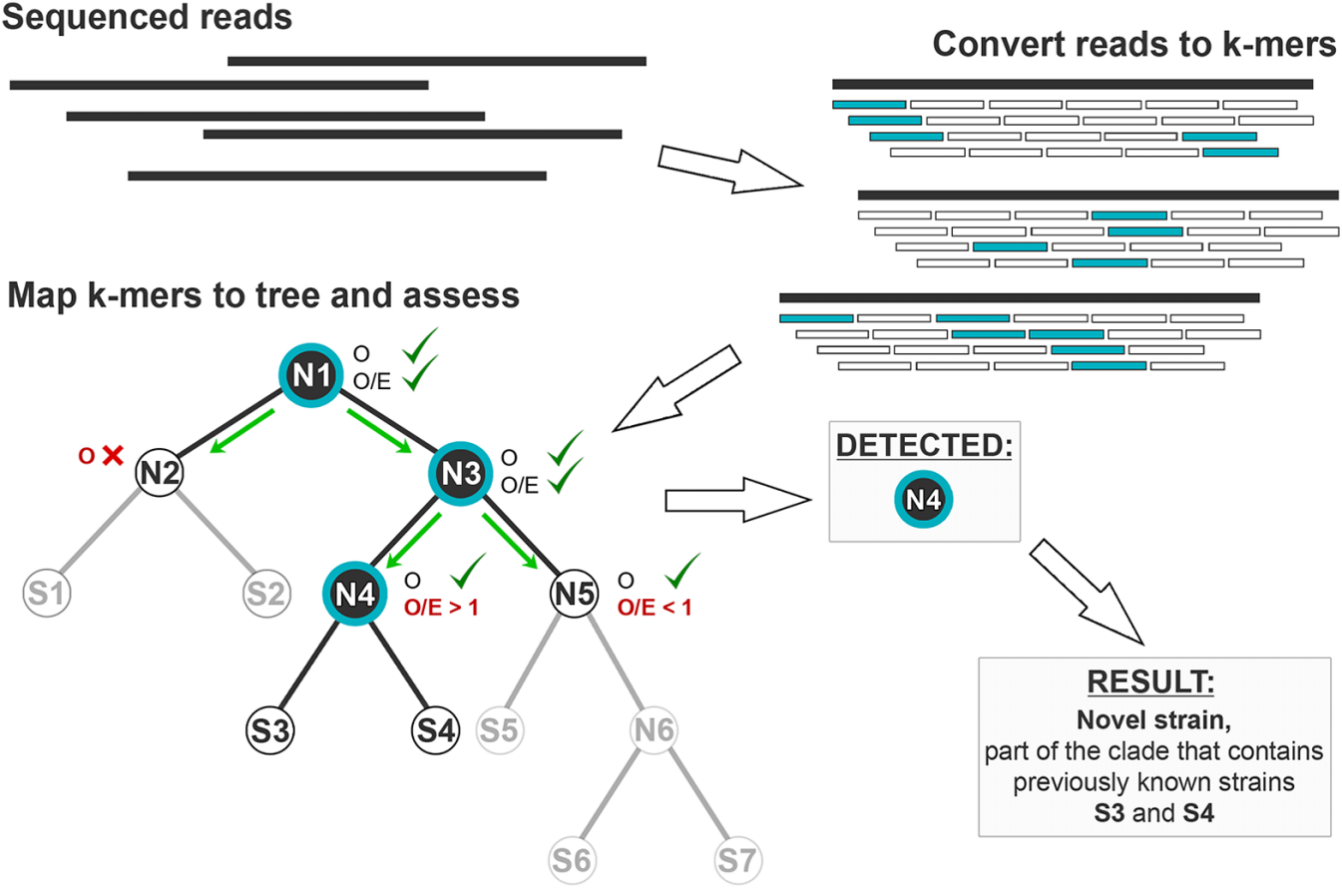
Strain identification process. After the sample is sequenced, the reads are converted into *k*-mers. Sample *k*-mers that are also present in the database (marked blue) are counted in their respective nodes (mapping to the tree). The search starts from root node (N1) and recursively moves down to the subnodes. First, the fraction of observed *k*-mers *O* is calculated (for details, see Methods). For the identification process to continue along the current branch, *O* is required to exceed a cutoff calculated for each node, otherwise the process stops (N2). Then, an observed/expected value *O*/*E* is calculated (see Methods) for nodes or the strain is shown as the result for the leaves. The process continues to the subnodes if *O*/*E* does not significantly differ from 1, showing that the current node is present in the sample and there is no branching to unknown nodes and strains. *O*/*E* values that are significantly higher than 1 (N4) indicate the presence of an unknown strain that belongs to the clade N4. *O*/*E* values that are significantly lower than 1 (N5) indicate possible sequencing errors and the process stops. The result above shows that a strain was found that belongs to the clade N4, which contains strains S3 and S4.

**Figure 3:**
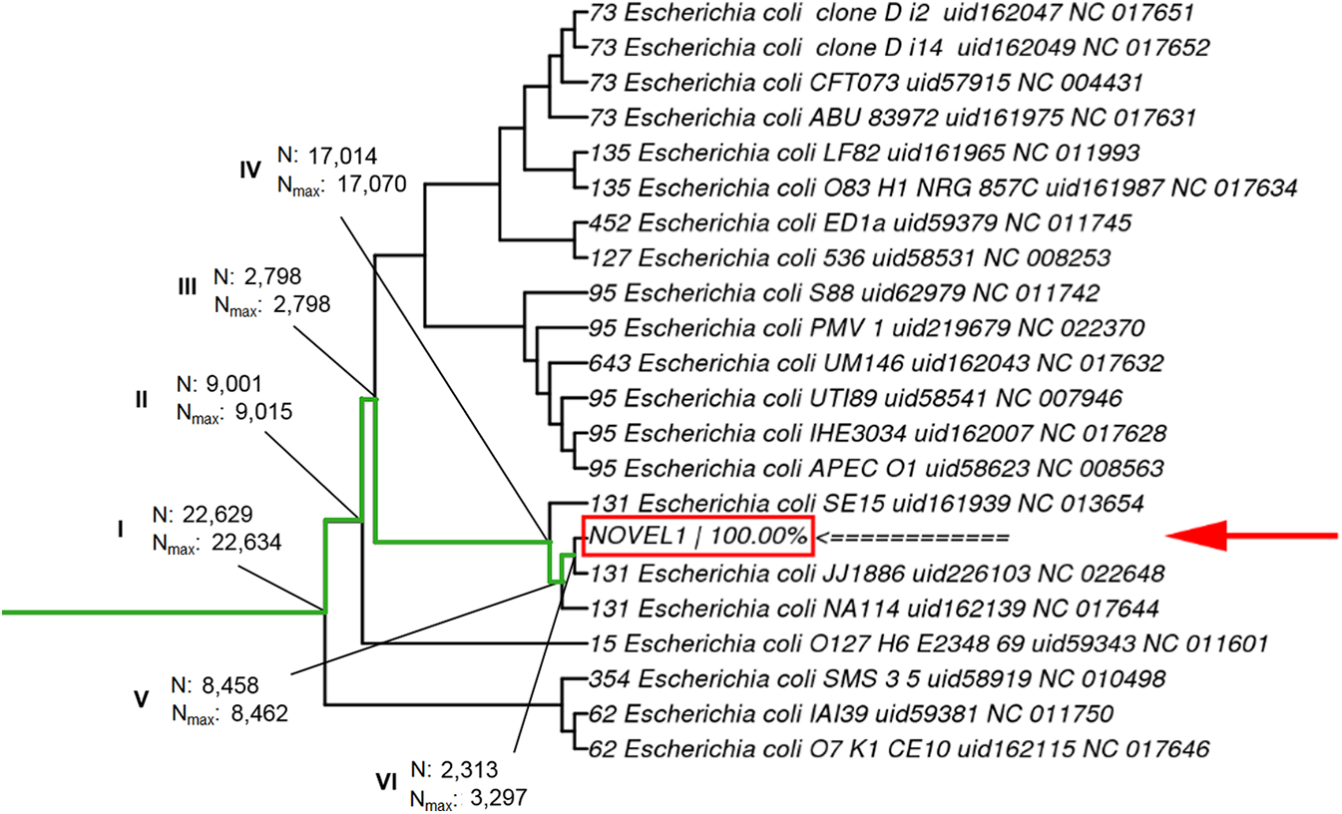
A visualized StrainSeeker result. The identified strain is marked with a red arrow and box. Percentage (100%) indicates that only this strain was found from the sample. Green line marks the path of StrainSeeker’s identification process, roman numerals mark the successive nodes that were present in the sample according to the algorithm. *N* indicates the number of *k*-mers specific to this node that were found in the sample, *N*_max_ indicates the maximum number of *k*-mers specific to the node. Each strain name is given as follows: [Multi-locus sequence type] [Strain name] [RefSeq identifier] [NCBI accession number]. The tree shown is a branch of the “Mash-based guide tree” (see Methods). The isolate in the example forms a small clade with the *Escherichia coli* strain JJ1886 and is marked “NOVEL,” indicating that it is a strain closely related to JJ1886, but not this exact strain. This is determined in step VI according to our algorithm (see Methods).

The search process follows the same guide tree structure. The search is recursive, starting the analysis of node-specific *k*-mers at the root node of the tree and moving down toward the potential newly characterized strains (Fig. 2). Depending on where the isolate branches off the guide tree, the result is given as a single strain or a clade (Fig. 3). In case of multiple strains present in the sample, all are reported with their respective fractions, which helps to detect contamination in the sample. StrainSeeker is implemented in PERL and can be run either as a stand-alone program on a UNIX server or as a web service. The output format of StrainSeeker is either a text file or a visualized result (Fig. S1A).

## Materials and Methods

### Building the guide trees

Four trees were built in total—three were used as guide trees for database building, one was used as a reference to determine the clades of 100 *E. coli* isolates used in testing.

**Figure 4:**
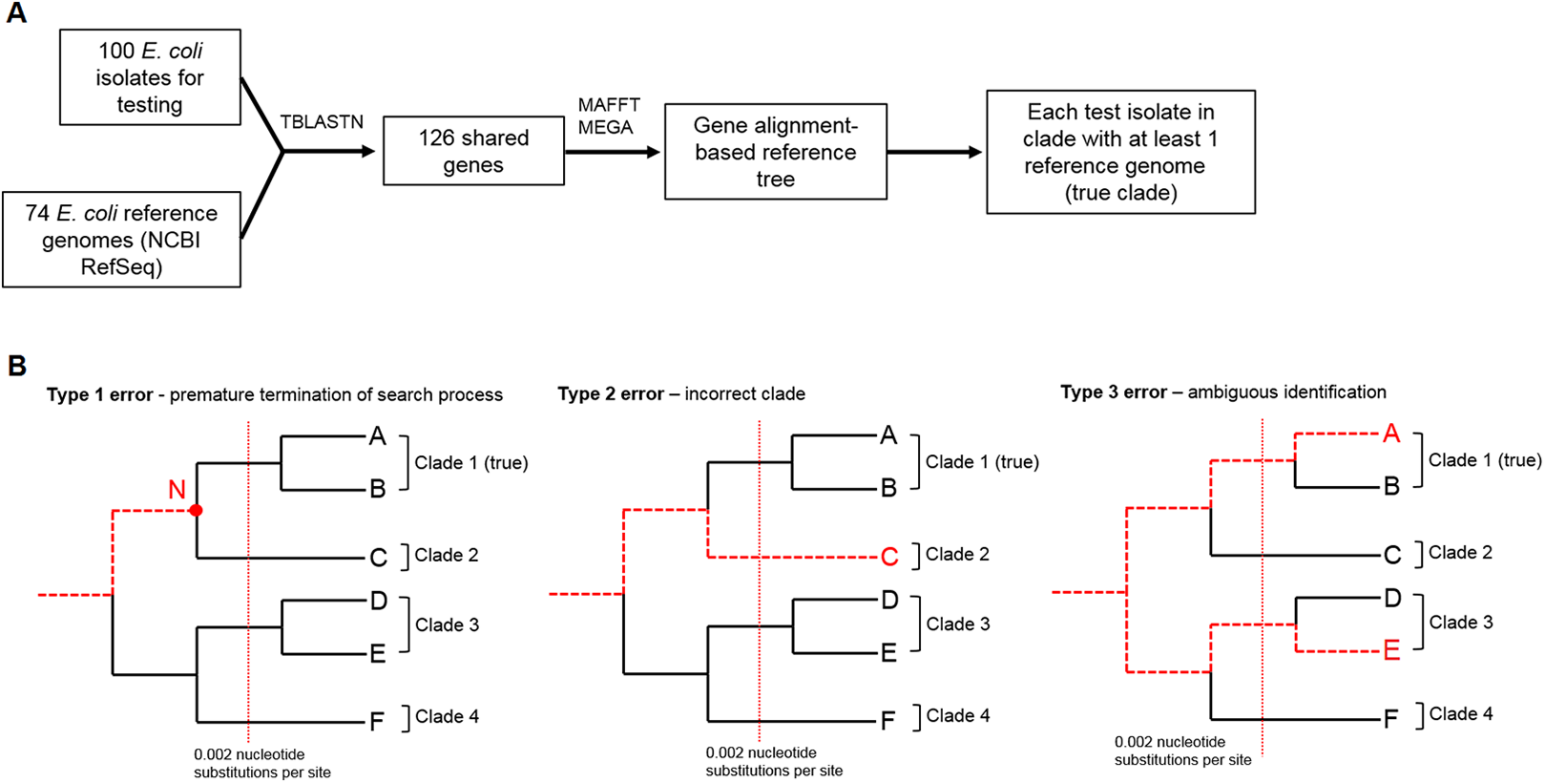
StrainSeeker testing workflow and error types. (A) The workflow that we used to build the “gene alignment-based reference tree” which was used to determine the true clade of each of the 100 *E. coli* isolates used in testing. Programs applied are marked on top of the arrows. (B) Three types of errors that StrainSeeker can make. Path of the search process is marked with a dashed red line. Type 1 error indicates that the search process stops (marked with N) before it reaches the correct clade and a larger clade containing Clade 1 and Clade 2 is reported. Type 2 error means that an incorrect clade (Clade 2) is reported instead of the true one (Clade 1). Type 3 error signifies an ambiguous result in which more than a single clade is reported (Clade 1 and additionally, Clade 3), each with a relative fraction in sample above 5%.

Two *E. coli* multiple gene alignment-based trees were built. First, the “gene alignment-based guide tree” contained 74 *E. coli* strains from the NCBI RefSeq database (release 69). Second, the “gene alignment-based reference tree” contained the same 74 strains from RefSeq and also 100 *E. coli* isolates that were used to test the performance of StrainSeeker (Table S1). We defined a clade by the phylogenetic distance—all the strains separated by less than 0.001 nucleotide substitutions per site were considered a clade (Fig. 4A). As the true strain-level identity of the isolates were not known, we used the “gene alignment-based reference tree” to determine the clades of the 100 isolates (Fig. S2). Similar phylogenetic trees have been used before for *E. coli* phylogenetic analysis (Ogura et al., 2009). Multiple alignments for both trees were built in a similar fashion. We extracted all *E. coli* genomic proteins from the UniProtKB/Swiss-Prot database (accessed 6/10/2016) and used TBLASTN 2.2.30 (Altschul et al., 1997) (match identity ≥90%, match coverage ≥95%) to check which proteins were present in each of the 174 *E. coli* genomes. The nucleotide sequences of 126 genes shared between all these strains (Table S2) were concatenated and a multiple alignment built with MAFFT v7.305b (parameters—*maxiterate 1000*) (Katoh et al., 2002). Trees were built with MEGA (Tamura et al., 2013), using neighbor-joining method and 500 bootstrap iterations.

The other two guide trees were built using a distance matrix made with an alignment-free, *k*-mer based method Mash (Ondov et al., 2016) (parameters *s* = 10,000, *k* = 21). The first, “Mash-based guide tree,” contained the same 74 *E. coli* strains as the “gene-based guide tree.” The other, “Large Mash-based guide tree,” contained all 4,324 available bacterial genomes from the NCBI RefSeq database (release 69). Trees were constructed with MEGA6 (Tamura et al., 2013) using the unweighted pair group method with arithmetic mean (UPGMA).

### StrainSeeker databases

We created all the databases using the GenomeTester4 software (Kaplinski, Lepamets & Remm, 2015). Nineteen databases with different *k*-mer lengths were built altogether, 14 contained 74 *E. coli* genomes obtained from the NCBI RefSeq database and were based on either the “gene alignment-based guide tree” or “Mash-based guide tree” (*k* ∈ *K*; *K* = {14, 15, 16, 20, 24, 28, 32}); five databases contained 4,324 bacterial genomes from the NCBI RefSeq database were based on the “Large Mash-based guide tree” (*k* ∈ *K*; *K* = {16, 20, 24, 28, 32}). Databases based on the “Large Mash-based guide tree” (*k < 16*) contained *E. coli* strains without any specific *k*-mers. These were omitted in the performance testing.

### StrainSeeker identification algorithm

First, the algorithm converts sequencing reads to a *k*-mer list (Fig. 2). Reads are converted to *k*-mers using a sliding window with a single nucleotide step. *K*-mers containing ambiguous nucleotides are removed. In the database, each guide tree node has a number of *k*-mers specific to it, referred to as “node-specific *k*-mers.”

The identification process starts at the root and recursively moves down toward the leaves. For each step, the percentage of observed *k*-mers *O* is calculated for the current node by dividing the number of node-specific *k*-mers *N* found in the sample with the total number of node-specific *k*-mers *N*_max_: *O = N/N*_max_. If *O* is below a minimum level, calculated based on the total number of *k*-mers in the node, the search will not continue further. Otherwise, an observed/expected ratio (*O*/*E*) of node-specific *k*-mers is calculated. The expected number of *k*-mers for the given node is the number of *k*-mers that should be observed, if bacteria from either of the two sub-clades of the node are present in sample. For a node A with children B and C it is as follows:

$$O/E = {O_{\rm{A}}} \div \left({{O_{\rm{B}}} + {O_{\rm{C}}}-{O_{\rm{B}}} \cdot {O_{\rm{C}}}} \right).$$

*O*/*E* < 1, indicates mainly sequencing errors; *O*/*E* > 1, indicates that that there is a strain that is related to the given node but not to either of its sub-nodes; *O*/*E* ≈ 1, indicates that at least one of the sub-nodes are present in the sample. We used an asymptotic test with a significance level of 5 × 10^−5^ to test the hypothesis that *O*/*E* = 1 (Article S1). If we cannot reject the hypothesis, the search will continue in sub-nodes with *O* and *O*/*E* calculated and checked at each step until either the strain level is reached or the hypothesis is rejected. If we reject the hypothesis, the current branch is either discarded because its apparent presence was due to noise (*O*/*E* < 1) or all strains under the current node will be reported in the output (*O*/*E* > 1).

To calculate the relative genome fractions in case of multiple strains present in the sample, we assumed that the number of times a *k*-mer is seen follows Poisson distribution. To reduce the influence of possible errors (either due to sequencing errors or a *k*-mer not being unique), we sorted the *k*-mer list by frequency and removed both the top 10% and the bottom 10% of *k*-mers from the list. We calculated the mean coverage from the remaining *k*-mers. Based on the mean of truncated observations, the mean of non-truncated Poisson distribution is estimated using the maximum-likelihood estimation.

### *Escherichia coli* and *Klebsiella pneumoniae* isolation, DNA sequencing and initial identification

*Escherichia coli* and *Klebsiella pneumoniae* strains used for testing were isolated from samples taken from hospitals during the project BARN. Full description and assembled sequences of the strains will be published elsewhere. Raw reads of all the strains used for testing are available at the European Nucleotide Archive (study accession PRJEB20419). The strains were isolated from different clinical materials: blood, pus, urine and the respiratory tract. Initial bacterial identification was performed using MALDI-TOF MS (Maldi Biotyper, BrukerDaltonics GmbH, Germany). DNA templates for sequencing were generated by growing isolate cultures overnight on blood agar (Oxoid Limited, Hampshire, UK). Total DNA from the bacterial strains was extracted using the QIAamp DNA Mini Kit (Qiagen, Hilden, Germany) and quantified using the Qubit® 2.0 Fluorometer (Invitrogen, Grand Island, NY, USA). A total of 1 ng of sample DNA was processed for the sequencing libraries using the Illumina Nextera XT sample preparation kit (Illumina, San Diego, CA, USA) according to the manufacturer’s instructions. The DNA normalization step was skipped; instead, the final dsDNA libraries were quantified with the Qubit® 2.0 Fluorometer and pooled in equimolar concentrations. The library pool was validated with 2200 TapeStation (Agilent Technologies, Santa Clara, CA, USA) measurements, and qPCR was performed with the Kapa Library Quantification Kit (Kapa Biosystems, Woburn, MA, USA) to optimize cluster generation. A total of 667 *E. coli* and 539 *K. pneumoniae* genomic libraries were sequenced with 2 × 101 base pair (bp) paired-end reads on the HiSeq2500 rapid run flowcell (Illumina, San Diego, CA, USA). Demultiplexing was performed with CASAVA 1.8.2. (Illumina, San Diego, CA, USA) allowing one mismatch in the index reads.

### *Escherichia coli* genome assembly and multi-locus sequence typing

Genomes were assembled with the *de novo* assembly program Velvet (Zerbino & Birney, 2008). Prior to assembling, the reads were trimmed and filtered for quality (*fastq_quality_trimmer–Q33–t 30–l 40, fastq_quality_filter–Q33–q 25–p 90*) (http://hannolab.cshl.edu/fastx_toolkit/). The cyclic assembly process was applied for each genome where different Velvet parameter values (*−exp_cov, −cov_cutoff (3, 5, 10, 15), −min_pair_count (1–5), −ins_length (100–350)*) were tested until all MLST genes were found or the best set of MLST genes was retrieved. For accurate MLST type identification, we used the assembled *E. coli* genomes and a MLST tool published by Larsen et al. (2012) that calculates the MLST profile based on a BLAST (Altschul et al., 1997) alignment of the input sequence file and the specified allele set. Public *E. coli* database “#1” version 2014_01 for molecular typing was downloaded from PubMLST (http://www.pubmlst.org/).

### Data sets used to assess the performance of StrainSeeker

We randomly selected 100 strains from 667 *E. coli* samples (Table S1). Assembled genomes of these strains were also included in the *E. coli* “gene alignment-based reference tree” (shown in Fig. S2) that we used to assess the results of StrainSeeker (Fig. 4).

In order to test the identification speed of the programs, we downloaded raw reads of three bacterial species from the European Nucleotide Archive (study accession PRJEB8647, run accession numbers ERR769199 [*Enterococcus faecium*], ERR769279 [*Enterococcus faecalis*] and ERR769315 [*Salmonella enterica*]) and also used raw reads of a randomly selected *K. pneumoniae* and *E. coli* isolate. Raw reads of 100 *E. coli* test strains and the *Klebsiella pneumoniae* isolate are available at the European Nucleotide Archive (study accession PRJEB20419).

## Results

### Using StrainSeeker to predict the clades of *E. coli* isolates

**Figure 5:**
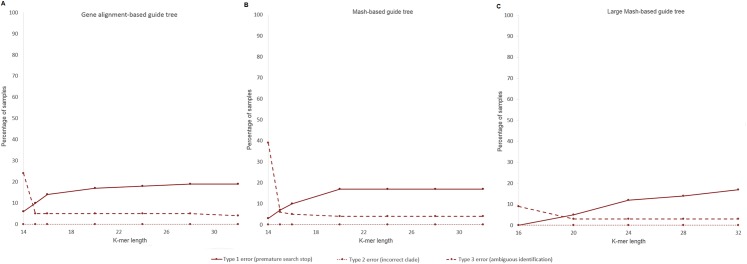
*K*-mer length effects on StrainSeeker results. A total of 100 *E. coli* samples were identified with StrainSeeker using databases based on three guide trees with *k*-mer lengths ranging from 14 to 32 (16 to 32 in case of (C), see Methods). (A) Identifications made with the database containing 74 *E. coli* strains. (B) Identifications made with the database containing 74 *E. coli* strains. (C) Identifications made with the 4,324 strains database.

To determine the correct clade for each of the 100 *E. coli* strains used in the test, we built the “gene alignment-based reference tree” (shown in Fig. S2) that included sequences of 100 test strains (Table S1) and sequences of 74 *E. coli* strains obtained from the NCBI RefSeq database. Clade threshold was set to 0.001 nucleotide substitutions per site. Tests run using databases based on three guide trees (see Methods)—the “gene-alignment-based guide tree” and the “Mash-based guide tree” contained 74 *E. coli* RefSeq strains (Figs. 5A and 5B) and the “Large Mash-based guide tree” contained 4,324 bacterial and archaeal strains obtained from the NCBI RefSeq database (Fig. 5C). We used the “gene alignment-based guide tree” as the positive control as we expected it to be the most accurate approximation of phylogenetic relationships. Versions with *k*-mer lengths 14–32 (16–32 in the case of the full NCBI bacteria database) were made from the databases. We counted the samples in which StrainSeeker made any of the three types of error (Fig. 4B). None of the isolates were assigned to incorrect clades. StrainSeeker’s ability to correctly predict the isolates’ clade increased with lower *k* values, mainly because its search process (Fig. 2) was more likely to stop prematurely in the case of higher *k*. However, in the case of *k* = 16 (*k* = 15 in the case of the small database), the number of ambiguous results increased. Therefore, shorter *k*-mers (16–20) are useful to avoid premature termination of the search process and longer *k*-mers (28–32) will prevent some of the ambiguous identifications. If a premature search stop is not a problem, longer *k*-mers can be used. StrainSeeker made more errors using the “gene alignment-based guide tree” compared to the “Mash-based guide tree,” in the case of *k* = 16 and higher values. Using the database based on the “Large Mash-based guide tree,” *k* = 16 and clade distance 0.001, StrainSeeker made less errors than with the other databases and its accuracy was 92% (Fig. 5C).

To see how well StrainSeeker can assign the clades of closely related *E. coli* strains, we tested the accuracy of StrainSeeker with smaller clades down to distance 0.00001, using the database based on the “Large Mash-based guide tree” and *k* = 16. This resulted in several clades in which the test strains were in a clade without any reference *E. coli* strains, which makes validation of these strains impossible. For this reason, the number of strains that can be validated decreases with decreasing the clade distance. With clade thresholds of 0.0003, 0.0001, 0.00003 and 0.00001 nucleotide substitutions per site, StrainSeeker identified the clades of 80 *E. coli* isolates with 90%, 69 isolates with 91%, 58 isolates with 90% and 46 isolates with 76% accuracy, respectively.

### Minimum amount of reads required for clade prediction

**Figure 6:**
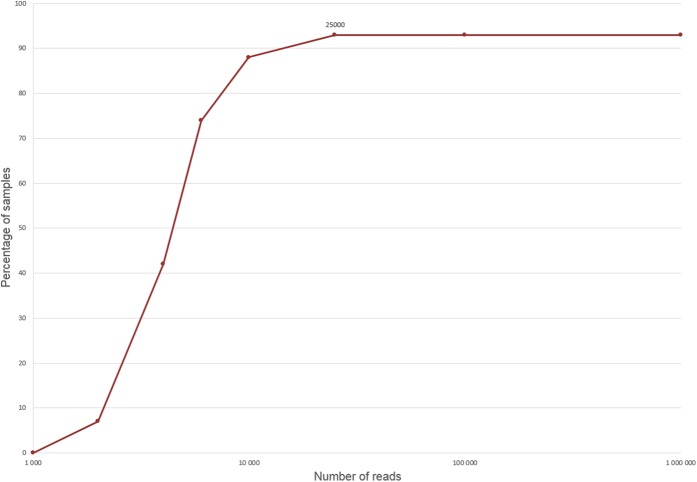
Minimum amount of reads required for isolate identification. The line shows the percentage of results without any types of error (Fig. 4B) obtained while sequencing a certain number of 101 bp Illumina reads. We used the large database, containing 4,324 strains, based on the “Large Mash-based guide tree” and *k* = 16.

To determine the required coverage for accurate isolate clade prediction using StrainSeeker, we used the same 100 *E. coli* test samples as above, but lowered the amount of reads analyzed. We used the large database, based on the “Large Mash-based guide tree” and *k* = 16. It can be seen that the number of results without any errors increases with the number of reads and the predictions are accurate if at least 25,000 reads from given strain are present (Fig. 6). This indicates that in order to predict the clade of an isolate, sequencing with low coverage is sufficient and many more samples could be sequenced simultaneously in a single sequencing run.

### StrainSeeker’s performance compared to other identification tools

We compared three other bacterial identification tools (Sigma, Reads2Type and Kraken) with StrainSeeker (Table 1). Kraken (Wood & Salzberg, 2014) classifies each sequencing read using exact *k*-mer matching, Sigma (Ahn, Chai & Pan, 2015) aligns reads to reference genomes and Reads2Type (Saputra et al., 2015) uses species-specific markers. Kraken and Sigma are designed to work on a UNIX server, Reads2Type is a web-based tool. All programs except Reads2Type were tested using a UNIX server, 1 CPU core and 512 GB total RAM.

**Table 1:** Speed comparison of StrainSeeker, Kraken, Sigma and Reads2Type. We used the “minikraken” database with Kraken, “fast” mode with Reads2Type and the 4,324 strain database based on the “Mash-based guide tree” and *k* = 16 in case of StrainSeeker.

Species	Read count (M)	Read length (bp)	Coverage	Identification time (min)			
				StrainSeeker	Sigma	Reads2Type	Kraken
*Escherichia coli*	1.39	101	28×	1.1	891.2	2.8	1.1
*Klebsiella pneumoniae*	2.37	101	46×	1.1	1303.6	3.3	1.7
*Enterococcus faecalis*	4.03	96	138×	1.1	2065.0	0.6	2.8
*Enterococcus faecium*	4.44	96	147×	1.1	2211.4	NA	3.0
*Salmonella enterica*	4.94	96	101×	1.2	2431.6	0.2	3.1

**Notes:**

Final size for the large, 4,324 strain database based on the “Mash-based guide tree” ranged from 4 to 18 GB (*k* = 16 and *k* = 32, respectively). If disk space for database building process is a constraint, databases can be downloaded from http://bioinfo.ut.ee/strainseeker or figshare (https://figshare.com/s/453ab0fb39ba6a06f91d). StrainSeeker does not load the whole database in memory and we successfully tested it using a laptop with 8 GB of RAM.

Accuracy was calculated based on the test set of 100 *E. coli* strains. All tools except Reads2Type were able to give multiple strains with different abundances as the output (Fig. S1), therefore it is not possible to use the same three types of errors as we used for describing StrainSeeker. Instead, we selected the strain with highest estimated abundance in the output file of each program and assessed whether it belonged to the correct clade or not. When measured by this method, we recorded the following results for identifying the correct strain in the set of 100 *E. coli* strains: StrainSeeker’s accuracy was 99% and Kraken’s accuracy 69%. Due to its excessive computing time (1,000× slower compared to other programs), we did not test the accuracy of Sigma. Reads2Type can identify samples at only species level (not at the strain level). Therefore, Reads2Type and Sigma were not used in this comparison.

Comparison of time spent on identification (Table 1) shows that Sigma spent several hours analyzing each sample, whereas the other tools took only a few minutes. This is mainly because read alignment is computationally more expensive than exact *k*-mer matching (Wood & Salzberg, 2014). Reads2Type identification speed varies and is not related to the sample size as it does not analyze all reads, but stops as soon as a read matches a unique probe. StrainSeeker scales well with large samples, taking almost the same amount of time for each sample. Identification results of all programs were correct on the species level, except that Reads2Type was unable to identify *Enterococcus faecium*.

## Discussion

It is paramount to identify novel strains because they can have very different phenotypes compared to their relatives. To solve this problem, we use a guide tree that allows us to narrow down the clade to which the isolate belongs. We decided not to use NCBI taxonomy because it did not contain strain-level relationships, making it unsuitable for the identification of clades within a species. Also in the NCBI tree, some taxons are not monophyletic, e.g., the *Shigella/Escherichia* branch.

Contrary to other *k*-mer-based programs identifying bacteria (Lindgreen, Adair & Gardner, 2016), StrainSeeker checks which of the specific *k*-mers are found in the sample, instead of each sequencing read. This makes StrainSeeker less vulnerable to errors if there are many *k*-mers in the sample (due to technical or biological reasons) which, according to the database, are specific to a species present in the database, but in fact, originate from another species not represented in the database. Also identifying each read separately could give a distorted result if the exact isolate is not present in the database. In such case, reads will be assigned to multiple bacterial genomes and the user cannot know if the sample contained an unknown strain or multiple related strains.

In the present work, we tested the performance of StrainSeeker using two different guide trees. One was based on an alignment of *E. coli* shared genes and included 74 *E. coli* strains, the other used a *k*-mer-based distance method (Ondov et al., 2016) and consisted of 4,324 bacterial and archaeal strains. StrainSeeker proved to be highly accurate in clade prediction especially with *k* values ranging from 16 to 20. Lower *k* values resulted in incorrect branches being identified along with the correct clade. This could be because of the sequencing errors as shorter *k*-mers are more likely assigned to wrong nodes due to errors than longer *k*-mers. Values for *k* higher than 20 are not recommended for clade prediction as StrainSeeker’s search process is more likely to stop prematurely. One reason for this is the total number of node-specific *k*-mers, which increases with higher *k* values as longer *k*-mers are more specific, but also more likely to contain sequencing errors.

In order to correctly predict phenotypic traits of an isolate, such as mutations conferring resistance to antibiotics, at least 10 times (10×) coverage is necessary (Inouye et al., 2014; Bradley et al., 2015). In our study, we demonstrated that the minimum amount of sequencing coverage required for accurate clade prediction is less than 1× in the case of *E. coli*. Based on this knowledge, multiple samples could be sequenced in a single run, saving resources and increasing throughput. This could be useful in all cases in which knowing only the clade of the strain would be sufficient, such as large-scale screening for known pathogenic bacteria.

Due to the statistical framework of StrainSeeker, it has some limitations. First, it is not able to differentiate between strains that are distinguished by only a few single nucleotide variations and may not be useful in detecting clinically relevant mutations and alleles. This requires high coverage and is a task more suited to tools like Mykrobe and SRST2. StrainSeeker is not meant to compete with such programs, but mainly to complement them. Second, only high-quality assembled genomes can be used as an input for StrainSeeker database building.

## Conclusion

There is a strong need for the fast detection of bacterial strains. StrainSeeker can detect strain sequences missing from public databases and identify the clade where the isolate belongs to. In the current study, we showed that StrainSeeker accurately and rapidly identifies the clades of 100 *E. coli* isolates. By using bacterial genome sequences from large public databases such as the NCBI RefSeq database, users do not have to build separate databases for each species of interest. Also StrainSeeker does not require high coverage for accurate clade prediction. For users who are not able to use the UNIX environment, there is an online version of StrainSeeker available at http://bioinfo.ut.ee/strainseeker/.

## Supplemental Information

10.7717/peerj.3353/supp-1 Supplemental Information 1 Output formats of StrainSeeker, Kraken, Sigma and Reads2Type. All tools were used to identify an *E. coli* isolate with multi-locus sequence type 131. According to our “gene alignment-based reference tree,” this strain was very similar to *E. coli* strain JJ1886. (**A**) StrainSeeker output is either given as a tab-delimited text or a pie chart with each strain relative abundance. Text format shows whether the identified strain was the same strain as the database reference strain (“KNOWN”) or related to it (“RELATED”). From the results, it can be seen that a single strain related to *E. coli* JJ1886 was found. (**B**) Kraken output is given as a tab-delimited text file with read numbers that were assigned to each taxonomic rank. *E. coli* JJ1886 is the strain with highest number of assigned reads, closely followed by O7:K1 which has the sequence type 62. (**C**) Sigma gives a html-format result which can be visualized in a web browser. *E. coli* JJ1886 has the highest percentage in the sample. (**D**) Reads2Type can only be used as a web tool and it gives a species-level result directly in the web browser.

10.7717/peerj.3353/supp-2 Supplemental Information 2 Gene alignment-based reference tree of *E. coli* strains. Each of the 74 NCBI RefSeq reference strain name is given as follows: [Multi-locus sequence type] [Strain name] [RefSeq identifier] [NCBI accession number]. The other 100 strains are the strains used in performance tests. The tree shown is the “gene alignment-based reference tree” (see Methods). Clades are limited by a maximum difference of 0.002 nucleotide substitutions per site between strains.

10.7717/peerj.3353/supp-3 Supplemental Information 3 *Escherichia coli* isolates used in the StrainSeeker performance tests.

10.7717/peerj.3353/supp-4 Supplemental Information 4 126 *E. coli* shared genes. A list of all the 126 protein-coding genes shared between all *E. coli* test isolates and reference E. coli strains.

10.7717/peerj.3353/supp-5 Supplemental Information 5 A thorough description of the statistical test that is part of the StrainSeeker identification algorithm. The description of the statistical test that is part of the StrainSeeker identification algorithm along with its derivation and all the notations used.

## List of abbreviations

bp: base pair
NCBI: National Center for Biotechnology Information
MLST: multi-locus sequence typing

## Additional Information and Declarations

### DNA Deposition

The following information was supplied regarding the deposition of DNA sequences: Raw reads of 100 *E. coli* test strains and the *Klebsiella pneumoniae* isolate are available at the European Nucleotide Archive (study accession PRJEB20419).

### Data Availability

The following information was supplied regarding data availability: The StrainSeeker code is available at both GitHub: https://github.com/bioinfo-ut and the department web server: http://bioinfo.ut.ee/strainseeker/. Roosaare, Märt (2017): StrainSeeker databases. figshare. https://doi.org/10.6084/m9.figshare.c.3750794.v1.
