# Supplementary figures and images for: StrainSeeker: fast identification of bacterial strains from raw sequencing reads using user-provided guide trees
Source: PeerJ. 2017 May 18;5:e3353. doi: 10.7717/peerj.3353 (PMC5438578; doi:10.7717/peerj.3353)

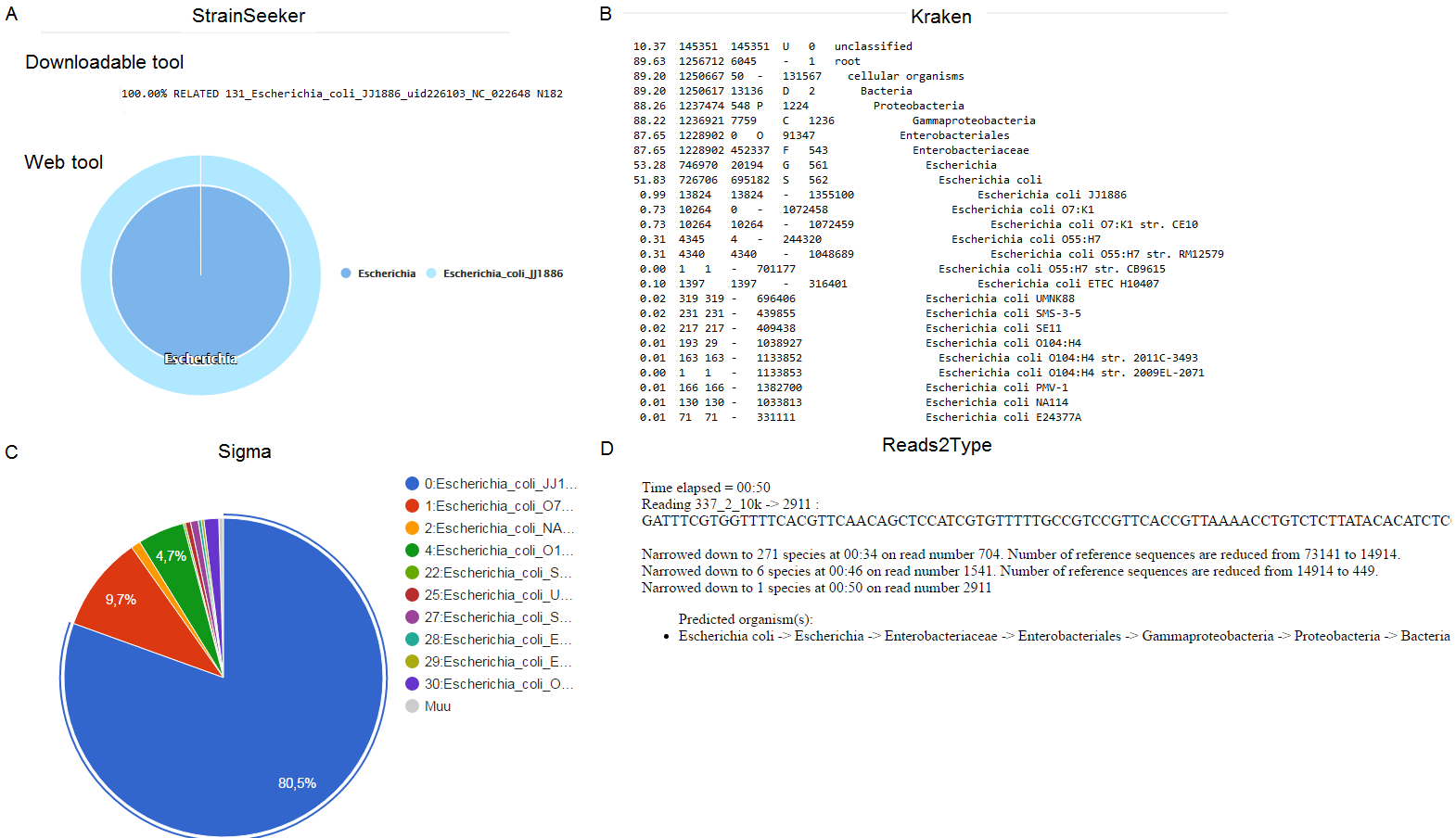

Supplement: Supplemental Information 1 — All tools were used to identify an E. coli isolate with multi-locus sequence type 131. According to our “gene alignment-based reference tree,” this strain was very similar to E. coli strain JJ1886. (A) StrainSeeker output is either given as a tab-delimited text or a pie chart with each strain relative abundance. Text format shows whether the identified strain was the same strain as the database reference strain (“KNOWN”) or related to it (“RELATED”). From the results, it can be seen that a single strain related to E. coli JJ1886 was found. (B) Kraken output is given as a tab-delimited text file with read numbers that were assigned to each taxonomic rank. E. coli JJ1886 is the strain with highest number of assigned reads, closely followed by O7:K1 which has the sequence type 62. (C) Sigma gives a html-format result which can be visualized in a web browser. E. coli JJ1886 has the highest percentage in the sample. (D) Reads2Type can only be used as a web tool and it gives a species-level result directly in the web browser. [file peerj-05-3353-s001.png]

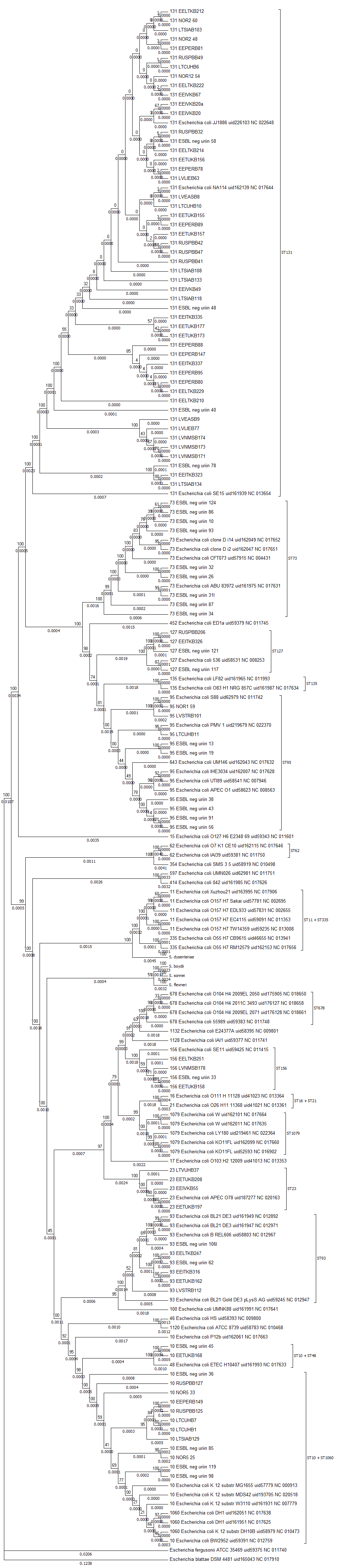

Supplement: Supplemental Information 2 — Each of the 74 NCBI RefSeq reference strain name is given as follows: [Multi-locus sequence type] [Strain name] [RefSeq identifier] [NCBI accession number]. The other 100 strains are the strains used in performance tests. The tree shown is the “gene alignment-based reference tree” (see Methods). Clades are limited by a maximum difference of 0.002 nucleotide substitutions per site between strains. [file peerj-05-3353-s002.png]
