# Supplementary material for: StrainSeeker: fast identification of bacterial strains from raw sequencing reads using user-provided guide trees
Source: PeerJ. 2017 May 18;5:e3353. doi: 10.7717/peerj.3353 (PMC5438578; doi:10.7717/peerj.3353)
Supplement: Supplemental Information 3 [file peerj-05-3353-s003.docx]

| **Species** | **N (isolates)** | **sequence type** |
| --- | --- | --- |
| *Escherichia coli* | 14 | ST10 |
|  | 4 | ST23 |
|  | 8 | ST73 |
|  | 6 | ST93 |
|  | 9 | ST95 |
|  | 4 | ST127 |
|  | 51 | ST131 |
|  | 4 | ST156 |
