# Supplementary material for: StrainSeeker: fast identification of bacterial strains from raw sequencing reads using user-provided guide trees
Source: PeerJ. 2017 May 18;5:e3353. doi: 10.7717/peerj.3353 (PMC5438578; doi:10.7717/peerj.3353)
Supplement: Supplemental Information 4 — A list of all the 126 protein-coding genes shared between all E. coli test isolates and reference E. coli strains. [file peerj-05-3353-s004.docx]

>sp|P00935|METB_ECOLI Cystathionine gamma-synthase OS=Escherichia coli (strain K12) GN=metB PE=1 SV=1

>sp|P05055|PNP_ECOLI Polyribonucleotide nucleotidyltransferase OS=Escherichia coli (strain K12) GN=pnp PE=1 SV=3

>sp|P07109|HISP_ECOLI Histidine transport ATP-binding protein HisP OS=Escherichia coli (strain K12) GN=hisP PE=1 SV=2

>sp|P09980|REP_ECOLI ATP-dependent DNA helicase Rep OS=Escherichia coli (strain K12) GN=rep PE=1 SV=3

>sp|P0A6C5|ARGA_ECOLI Amino-acid acetyltransferase OS=Escherichia coli (strain K12) GN=argA PE=1 SV=1

>sp|P0A6E4|ASSY_ECOLI Argininosuccinate synthase OS=Escherichia coli (strain K12) GN=argG PE=1 SV=2

>sp|P0A6E6|ATPE_ECOLI ATP synthase epsilon chain OS=Escherichia coli (strain K12) GN=atpC PE=1 SV=2

>sp|P0A6F5|CH60_ECOLI 60 kDa chaperonin OS=Escherichia coli (strain K12) GN=groL PE=1 SV=2

>sp|P0A6I0|KCY_ECOLI Cytidylate kinase OS=Escherichia coli (strain K12) GN=cmk PE=1 SV=1

>sp|P0A6L0|DEOC_ECOLI Deoxyribose-phosphate aldolase OS=Escherichia coli (strain K12) GN=deoC PE=1 SV=1

>sp|P0A6N4|EFP_ECOLI Elongation factor P OS=Escherichia coli (strain K12) GN=efp PE=1 SV=2

>sp|P0A6R3|FIS_ECOLI DNA-binding protein Fis OS=Escherichia coli (strain K12) GN=fis PE=1 SV=1

>sp|P0A6T1|G6PI_ECOLI Glucose-6-phosphate isomerase OS=Escherichia coli (strain K12) GN=pgi PE=1 SV=1

>sp|P0A6W5|GREA_ECOLI Transcription elongation factor GreA OS=Escherichia coli (strain K12) GN=greA PE=1 SV=1

>sp|P0A6X7|IHFA_ECOLI Integration host factor subunit alpha OS=Escherichia coli (strain K12) GN=ihfA PE=1 SV=1

>sp|P0A717|KPRS_ECOLI Ribose-phosphate pyrophosphokinase OS=Escherichia coli (strain K12) GN=prs PE=1 SV=2

>sp|P0A766|RSXA_ECOLI Electron transport complex subunit RsxA OS=Escherichia coli (strain K12) GN=rsxA PE=1 SV=1

>sp|P0A780|NUSB_ECOLI N utilization substance protein B OS=Escherichia coli (strain K12) GN=nusB PE=1 SV=1

>sp|P0A7C2|LEXA_ECOLI LexA repressor OS=Escherichia coli (strain K12) GN=lexA PE=1 SV=1

>sp|P0A7G2|RBFA_ECOLI Ribosome-binding factor A OS=Escherichia coli (strain K12) GN=rbfA PE=1 SV=2

>sp|P0A7G6|RECA_ECOLI Protein RecA OS=Escherichia coli (strain K12) GN=recA PE=1 SV=2

>sp|P0A7I4|RF3_ECOLI Peptide chain release factor 3 OS=Escherichia coli (strain K12) GN=prfC PE=1 SV=2

>sp|P0A7L8|RL27_ECOLI 50S ribosomal protein L27 OS=Escherichia coli (strain K12) GN=rpmA PE=1 SV=2

>sp|P0A7M2|RL28_ECOLI 50S ribosomal protein L28 OS=Escherichia coli (strain K12) GN=rpmB PE=1 SV=2

>sp|P0A7M6|RL29_ECOLI 50S ribosomal protein L29 OS=Escherichia coli (strain K12) GN=rpmC PE=1 SV=1

>sp|P0A7S3|RS12_ECOLI 30S ribosomal protein S12 OS=Escherichia coli (strain K12) GN=rpsL PE=1 SV=2

>sp|P0A7T7|RS18_ECOLI 30S ribosomal protein S18 OS=Escherichia coli (strain K12) GN=rpsR PE=1 SV=2

>sp|P0A7U3|RS19_ECOLI 30S ribosomal protein S19 OS=Escherichia coli (strain K12) GN=rpsS PE=1 SV=2

>sp|P0A7W7|RS8_ECOLI 30S ribosomal protein S8 OS=Escherichia coli (strain K12) GN=rpsH PE=1 SV=2

>sp|P0A7Z4|RPOA_ECOLI DNA-directed RNA polymerase subunit alpha OS=Escherichia coli (strain K12) GN=rpoA PE=1 SV=1

>sp|P0A805|RRF_ECOLI Ribosome-recycling factor OS=Escherichia coli (strain K12) GN=frr PE=1 SV=1

>sp|P0A814|RUVC_ECOLI Crossover junction endodeoxyribonuclease RuvC OS=Escherichia coli (strain K12) GN=ruvC PE=1 SV=2

>sp|P0A825|GLYA_ECOLI Serine hydroxymethyltransferase OS=Escherichia coli (strain K12) GN=glyA PE=1 SV=1

>sp|P0A836|SUCC_ECOLI Succinate--CoA ligase [ADP-forming] subunit beta OS=Escherichia coli (strain K12) GN=sucC PE=1 SV=1

>sp|P0A879|TRPB_ECOLI Tryptophan synthase beta chain OS=Escherichia coli (strain K12) GN=trpB PE=1 SV=2

>sp|P0A8F4|URK_ECOLI Uridine kinase OS=Escherichia coli (strain K12) GN=udk PE=3 SV=1

>sp|P0A8R0|RRAA_ECOLI Regulator of ribonuclease activity A OS=Escherichia coli (strain K12) GN=rraA PE=1 SV=1

>sp|P0A8V2|RPOB_ECOLI DNA-directed RNA polymerase subunit beta OS=Escherichia coli (strain K12) GN=rpoB PE=1 SV=1

>sp|P0A912|PAL_ECOLI Peptidoglycan-associated lipoprotein OS=Escherichia coli (strain K12) GN=pal PE=1 SV=1

>sp|P0A9B2|G3P1_ECOLI Glyceraldehyde-3-phosphate dehydrogenase A OS=Escherichia coli (strain K12) GN=gapA PE=1 SV=2

>sp|P0A9D8|DAPD_ECOLI 2,3,4,5-tetrahydropyridine-2,6-dicarboxylate N-succinyltransferase OS=Escherichia coli (strain K12) GN=dapD PE=1 SV=1

>sp|P0A9E5|FNR_ECOLI Fumarate and nitrate reduction regulatory protein OS=Escherichia coli (strain K12) GN=fnr PE=1 SV=1

>sp|P0A9M8|PTA_ECOLI Phosphate acetyltransferase OS=Escherichia coli (strain K12) GN=pta PE=1 SV=2

>sp|P0A9N4|PFLA_ECOLI Pyruvate formate-lyase 1-activating enzyme OS=Escherichia coli (strain K12) GN=pflA PE=1 SV=2

>sp|P0A9U3|YBIT_ECOLI Uncharacterized ABC transporter ATP-binding protein YbiT OS=Escherichia coli (strain K12) GN=ybiT PE=1 SV=1

>sp|P0AA04|PTHP_ECOLI Phosphocarrier protein HPr OS=Escherichia coli (strain K12) GN=ptsH PE=1 SV=1

>sp|P0AA10|RL13_ECOLI 50S ribosomal protein L13 OS=Escherichia coli (strain K12) GN=rplM PE=1 SV=1

>sp|P0AA25|THIO_ECOLI Thioredoxin-1 OS=Escherichia coli (strain K12) GN=trxA PE=1 SV=2

>sp|P0AAF6|ARTP_ECOLI Arginine transport ATP-binding protein ArtP OS=Escherichia coli (strain K12) GN=artP PE=1 SV=1

>sp|P0AAZ4|RARA_ECOLI Replication-associated recombination protein A OS=Escherichia coli (strain K12) GN=rarA PE=1 SV=1

>sp|P0AB89|PUR8_ECOLI Adenylosuccinate lyase OS=Escherichia coli (strain K12) GN=purB PE=1 SV=1

>sp|P0ABA0|ATPF_ECOLI ATP synthase subunit b OS=Escherichia coli (strain K12) GN=atpF PE=1 SV=1

>sp|P0ABA6|ATPG_ECOLI ATP synthase gamma chain OS=Escherichia coli (strain K12) GN=atpG PE=1 SV=1

>sp|P0ABH0|FTSA_ECOLI Cell division protein FtsA OS=Escherichia coli (strain K12) GN=ftsA PE=1 SV=1

>sp|P0ABT2|DPS_ECOLI DNA protection during starvation protein OS=Escherichia coli (strain K12) GN=dps PE=1 SV=2

>sp|P0ABT5|DUSB_ECOLI tRNA-dihydrouridine synthase B OS=Escherichia coli (strain K12) GN=dusB PE=3 SV=1

>sp|P0ABU9|TOLQ_ECOLI Protein TolQ OS=Escherichia coli (strain K12) GN=tolQ PE=1 SV=1

>sp|P0AC02|BAMD_ECOLI Outer membrane protein assembly factor BamD OS=Escherichia coli (strain K12) GN=bamD PE=1 SV=1

>sp|P0AC38|ASPA_ECOLI Aspartate ammonia-lyase OS=Escherichia coli (strain K12) GN=aspA PE=1 SV=1

>sp|P0AC41|SDHA_ECOLI Succinate dehydrogenase flavoprotein subunit OS=Escherichia coli (strain K12) GN=sdhA PE=1 SV=1

>sp|P0AC53|G6PD_ECOLI Glucose-6-phosphate 1-dehydrogenase OS=Escherichia coli (strain K12) GN=zwf PE=1 SV=1

>sp|P0AC55|GLNK_ECOLI Nitrogen regulatory protein P-II 2 OS=Escherichia coli (strain K12) GN=glnK PE=1 SV=1

>sp|P0AC81|LGUL_ECOLI Lactoylglutathione lyase OS=Escherichia coli (strain K12) GN=gloA PE=1 SV=1

>sp|P0ACI6|ASNC_ECOLI Regulatory protein AsnC OS=Escherichia coli (strain K12) GN=asnC PE=1 SV=1

>sp|P0ACJ0|LRP_ECOLI Leucine-responsive regulatory protein OS=Escherichia coli (strain K12) GN=lrp PE=1 SV=2

>sp|P0ACP5|GNTR_ECOLI HTH-type transcriptional regulator GntR OS=Escherichia coli (strain K12) GN=gntR PE=1 SV=1

>sp|P0ACU5|FABR_ECOLI HTH-type transcriptional repressor FabR OS=Escherichia coli (strain K12) GN=fabR PE=1 SV=1

>sp|P0ADY7|RL16_ECOLI 50S ribosomal protein L16 OS=Escherichia coli (strain K12) GN=rplP PE=1 SV=1

>sp|P0ADZ4|RS15_ECOLI 30S ribosomal protein S15 OS=Escherichia coli (strain K12) GN=rpsO PE=1 SV=2

>sp|P0AE34|ARTQ_ECOLI Arginine ABC transporter permease protein ArtQ OS=Escherichia coli (strain K12) GN=artQ PE=1 SV=1

>sp|P0AE52|BCP_ECOLI Putative peroxiredoxin bcp OS=Escherichia coli (strain K12) GN=bcp PE=1 SV=1

>sp|P0AE88|CPXR_ECOLI Transcriptional regulatory protein CpxR OS=Escherichia coli (strain K12) GN=cpxR PE=1 SV=1

>sp|P0AEI1|MIAB_ECOLI tRNA-2-methylthio-N(6)-dimethylallyladenosine synthase OS=Escherichia coli (strain K12) GN=miaB PE=1 SV=1

>sp|P0AEJ4|ENVZ_ECOLI Osmolarity sensor protein EnvZ OS=Escherichia coli (strain K12) GN=envZ PE=1 SV=1

>sp|P0AEK4|FABI_ECOLI Enoyl-[acyl-carrier-protein] reductase [NADH] FabI OS=Escherichia coli (strain K12) GN=fabI PE=1 SV=2

>sp|P0AEQ6|GLNP_ECOLI Glutamine transport system permease protein GlnP OS=Escherichia coli (strain K12) GN=glnP PE=1 SV=1

>sp|P0AEU3|HISM_ECOLI Histidine transport system permease protein HisM OS=Escherichia coli (strain K12) GN=hisM PE=1 SV=1

>sp|P0AEW9|K1PF_ECOLI 1-phosphofructokinase OS=Escherichia coli (strain K12) GN=fruK PE=3 SV=1

>sp|P0AEZ1|METF_ECOLI 5,10-methylenetetrahydrofolate reductase OS=Escherichia coli (strain K12) GN=metF PE=1 SV=1

>sp|P0AF16|MURJ_ECOLI Lipid II flippase MurJ OS=Escherichia coli (strain K12) GN=murJ PE=1 SV=1

>sp|P0AFB1|NLPI_ECOLI Lipoprotein NlpI OS=Escherichia coli (strain K12) GN=nlpI PE=1 SV=1

>sp|P0AFB8|NTRC_ECOLI Nitrogen regulation protein NR(I) OS=Escherichia coli (strain K12) GN=glnG PE=1 SV=1

>sp|P0AFD4|NUOH_ECOLI NADH-quinone oxidoreductase subunit H OS=Escherichia coli (strain K12) GN=nuoH PE=1 SV=1

>sp|P0AFD6|NUOI_ECOLI NADH-quinone oxidoreductase subunit I OS=Escherichia coli (strain K12) GN=nuoI PE=1 SV=1

>sp|P0AFE4|NUOK_ECOLI NADH-quinone oxidoreductase subunit K OS=Escherichia coli (strain K12) GN=nuoK PE=1 SV=1

>sp|P0AFF6|NUSA_ECOLI Transcription termination/antitermination protein NusA OS=Escherichia coli (strain K12) GN=nusA PE=1 SV=1

>sp|P0AFG3|ODO1_ECOLI 2-oxoglutarate dehydrogenase E1 component OS=Escherichia coli (strain K12) GN=sucA PE=1 SV=1

>sp|P0AFG8|ODP1_ECOLI Pyruvate dehydrogenase E1 component OS=Escherichia coli (strain K12) GN=aceE PE=1 SV=2

>sp|P0AFN6|YADH_ECOLI Inner membrane transport permease YadH OS=Escherichia coli (strain K12) GN=yadH PE=1 SV=1

>sp|P0AFZ7|TRKH_ECOLI Trk system potassium uptake protein TrkH OS=Escherichia coli (strain K12) GN=trkH PE=1 SV=1

>sp|P0AG59|RS14_ECOLI 30S ribosomal protein S14 OS=Escherichia coli (strain K12) GN=rpsN PE=1 SV=2

>sp|P0AG67|RS1_ECOLI 30S ribosomal protein S1 OS=Escherichia coli (strain K12) GN=rpsA PE=1 SV=1

>sp|P0AGB6|RPOE_ECOLI ECF RNA polymerase sigma-E factor OS=Escherichia coli (strain K12) GN=rpoE PE=1 SV=1

>sp|P0AGE9|SUCD_ECOLI Succinate--CoA ligase [ADP-forming] subunit alpha OS=Escherichia coli (strain K12) GN=sucD PE=1 SV=2

>sp|P0AGJ7|TRML_ECOLI tRNA (cytidine(34)-2'-O)-methyltransferase OS=Escherichia coli (strain K12) GN=trmL PE=1 SV=1

>sp|P0AGM7|URAA_ECOLI Uracil permease OS=Escherichia coli (strain K12) GN=uraA PE=1 SV=1

>sp|P0C054|IBPA_ECOLI Small heat shock protein IbpA OS=Escherichia coli (strain K12) GN=ibpA PE=1 SV=1

>sp|P0C0L9|ISCX_ECOLI Protein IscX OS=Escherichia coli (strain K12) GN=iscX PE=1 SV=1

>sp|P0C0R7|RLME_ECOLI Ribosomal RNA large subunit methyltransferase E OS=Escherichia coli (strain K12) GN=rlmE PE=1 SV=1

>sp|P0CG19|RNPH_ECOLI Inactive ribonuclease PH OS=Escherichia coli (strain K12) GN=rph PE=3 SV=1

>sp|P15993|AROP_ECOLI Aromatic amino acid transport protein AroP OS=Escherichia coli (strain K12) GN=aroP PE=1 SV=3

>sp|P16659|SYP_ECOLI Proline--tRNA ligase OS=Escherichia coli (strain K12) GN=proS PE=1 SV=4

>sp|P16701|CYST_ECOLI Sulfate transport system permease protein CysT OS=Escherichia coli (strain K12) GN=cysU PE=3 SV=1

>sp|P21170|SPEA_ECOLI Biosynthetic arginine decarboxylase OS=Escherichia coli (strain K12) GN=speA PE=1 SV=2

>sp|P22731|LIVF_ECOLI High-affinity branched-chain amino acid transport ATP-binding protein LivF OS=Escherichia coli (strain K12) GN=livF PE=1 SV=2

>sp|P23894|HTPX_ECOLI Protease HtpX OS=Escherichia coli (strain K12) GN=htpX PE=1 SV=1

>sp|P25522|MNME_ECOLI tRNA modification GTPase MnmE OS=Escherichia coli (strain K12) GN=mnmE PE=1 SV=3

>sp|P33599|NUOCD_ECOLI NADH-quinone oxidoreductase subunit C/D OS=Escherichia coli (strain K12) GN=nuoC PE=1 SV=3

>sp|P36938|PGM_ECOLI Phosphoglucomutase OS=Escherichia coli (strain K12) GN=pgm PE=1 SV=1

>sp|P37773|MPL_ECOLI UDP-N-acetylmuramate--L-alanyl-gamma-D-glutamyl-meso-2,6-diaminoheptandioate ligase OS=Escherichia coli (strain K12) GN=mpl PE=1 SV=3

>sp|P60723|RL4_ECOLI 50S ribosomal protein L4 OS=Escherichia coli (strain K12) GN=rplD PE=1 SV=1

>sp|P61175|RL22_ECOLI 50S ribosomal protein L22 OS=Escherichia coli (strain K12) GN=rplV PE=1 SV=1

>sp|P61517|CAN_ECOLI Carbonic anhydrase 2 OS=Escherichia coli (strain K12) GN=can PE=1 SV=1

>sp|P62617|ISPF_ECOLI 2-C-methyl-D-erythritol 2,4-cyclodiphosphate synthase OS=Escherichia coli (strain K12) GN=ispF PE=1 SV=1

>sp|P62620|ISPG_ECOLI 4-hydroxy-3-methylbut-2-en-1-yl diphosphate synthase (flavodoxin) OS=Escherichia coli (strain K12) GN=ispG PE=1 SV=1

>sp|P63177|RLMB_ECOLI 23S rRNA (guanosine-2'-O-)-methyltransferase RlmB OS=Escherichia coli (strain K12) GN=rlmB PE=1 SV=1

>sp|P65870|QUED_ECOLI 6-carboxy-5,6,7,8-tetrahydropterin synthase OS=Escherichia coli (strain K12) GN=queD PE=1 SV=1

>sp|P66817|DIAA_ECOLI DnaA initiator-associating protein DiaA OS=Escherichia coli (strain K12) GN=diaA PE=1 SV=1

>sp|P67910|HLDD_ECOLI ADP-L-glycero-D-manno-heptose-6-epimerase OS=Escherichia coli (strain K12) GN=hldD PE=1 SV=1

>sp|P68699|ATPL_ECOLI ATP synthase subunit c OS=Escherichia coli (strain K12) GN=atpE PE=1 SV=1

>sp|P69222|IF1_ECOLI Translation initiation factor IF-1 OS=Escherichia coli (strain K12) GN=infA PE=1 SV=2

>sp|P69441|KAD_ECOLI Adenylate kinase OS=Escherichia coli (strain K12) GN=adk PE=1 SV=1

>sp|P69776|LPP_ECOLI Major outer membrane lipoprotein Lpp OS=Escherichia coli (strain K12) GN=lpp PE=1 SV=1

>sp|P69924|RIR2_ECOLI Ribonucleoside-diphosphate reductase 1 subunit beta OS=Escherichia coli (strain K12) GN=nrdB PE=1 SV=2

>sp|P69931|HDA_ECOLI DnaA regulatory inactivator Hda OS=Escherichia coli (strain K12) GN=hda PE=1 SV=2

>sp|P75825|HCP_ECOLI Hydroxylamine reductase OS=Escherichia coli (strain K12) GN=hcp PE=1 SV=2
