# Supplementary material for: StrainSeeker: fast identification of bacterial strains from raw sequencing reads using user-provided guide trees
Source: PeerJ. 2017 May 18;5:e3353. doi: 10.7717/peerj.3353 (PMC5438578; doi:10.7717/peerj.3353)
Supplement: Supplemental Information 5 — The description of the statistical test that is part of the StrainSeeker identification algorithm along with its derivation and all the notations used. [file peerj-05-3353-s005.pdf]

Ratio of Observed and Expected k-mer counts
Statistical properties

Märt Möls

June 8, 2015

Abstract

When counting species- (or strain-) specific k-mers the resulting number can vary due to randomness in placement of reads (if another run of reads is made from the same sample with the same coverage the resulting number of species-specific k-mers seen in the sample will be probably different from the result achieved in the first run). This variability is present wether one counts the k-mers specific to an interior node (k-mers present in all descendants of the node but not present in any other species in other branches of the phylogenetic tree) or counts the k-mers specific to the descendant of the node. This results also in variability in the Expected k-mer count, which is calculated under the assumption of existence of both descendants (in varying quantity) in the sample:

$$\hat{E} = (\hat{P}_1 + \hat{P}_2 - \hat{P}_1\hat{P}_2) \cdot \text{number of node specific k-mers},$$

where $\hat{E}$ denotes expected k-mer count of a node, $\hat{P}_1$ is the proportion of descendant 1 specific k-mers found in the sample and $\hat{P}_2$ is the proportion of k-mers specific to the descendant 2. In this report formulas for the variance of observed and expected k-mer counts, $\mathrm{Var}(O)$ and $\mathrm{Var}(\hat{E})$, are given; also the formula and it's derivation to calculate approximate (asymptotically correct) confidence intervals for observed/expected ratio is presented. Also an asymptotic test to test hypothesis about observed/expected ratio is proposed.

Chapter 1

Notations

O - number of unique k-mers seen in a random (next) sample;

- O_1 - number of k-mers specific to species 1 discovered;
- O_2 - number of k-mers specific to species 2 discovered;
- o, o_1, o_2 - number of unique k-mers seen (one or more times) in the sample;

n - total number of unique k-mers searched;

- n_1 - number of k-mers specific to species 1 searched for;
- n_2 - number of k-mers specific to species 2 searched for;
- n_{node} - number of k-mers specific to internal node searched for;

p_1, p_2 - probabilities to see a k-mer specific to species 1 and species 2 respectively.

$\hat{P}_1$, $\hat{P}_2$ - estimators for the probabilities p_1 and p_2: $\hat{P}_1 = O_1/n_1, \hat{P}_2 = O_2/n_2$.

$\hat{p}_1, \hat{p}_2$ - the realised values of $\hat{P}_1$ and $\hat{P}_2$ in the current sample.

l - read length;

k - length of k-mer;

S_i - number of reads starting from base i;

I_i - indicator variable: $I_i = 1$, if k-mer starting from position i in genome cannot be found in reads; $I_i = 0$ if the k-mer has been seen one or more times.

e - Excpected number of node-specific k-mers discovered, based on the number of species-specific k-mers seen ($e = p_1 + p_2 - p_1 p_2$)

$\hat{E}$ - estimator for E, $\hat{E} = \hat{P}_1 + \hat{P}_2 - \hat{P}_1 \cdot \hat{P}_2$. Random variable.

$\hat{e}$ - the value of $\hat{E}$ in the current sample. Constant.

Chapter 2

Variance for the number of (unique) k-mers seen

2.1 Formulas

$$\hat{\lambda} = -\ln\left(1 - o/n\right)/(l - k + 1) \tag{2.1}$$

$$\widehat{\mathrm{Var}(O)} = o(1 - o/n) + \sum_{j=1}^{\min(l-k,n-1)} 2(n-j)\Big\{\exp(-\hat{\lambda}(l-k+1+j)) - \exp(-2\hat{\lambda}(l-k+1))\Big\} \tag{2.2}$$

$$\widehat{\mathrm{Var}(\hat{E})} = n_{node}^2 \left(\frac{\widehat{\mathrm{Var}(O_1)}}{n_1^2}[1 + \widehat{\mathrm{Var}(O_2)}/n_2^2 + (o_2/n_2)^2 - 2o_2/n_2] + \frac{\widehat{\mathrm{Var}(O_2)}}{n_2^2}[1 + (o_1/n_1)^2 - 2o_1/n_1]\right). \tag{2.3}$$

These formulas are derived under worst-case scenario — by assuming the unique k-mers to be located next to each other (next base in genome is also the starting point of a next k-mer in k-mer search list). If the unique k-mers are separated from each other then the variance of O is smaller than the number given by the formula (2.2) and the variance of $\hat{E}$ is smaller than the value calculated by (2.3).

2.2 Derivation

We assume the number of reads starting from a base i in genome to have Poisson distribution,

$$S_i \sim Poi(\lambda).$$

Similar assumptions are also made in Illumina technical materials, see for example [1].

One unique k-mer starting from position i is seen exactly $K_i := S_i + S_{i-1} + \ldots + S_{i-l+k}$ times. Because the starting points of reads can be assumed to be independent of each other

$$K_i \sim Poi\left(\lambda[l-k+1]\right),$$

because the sum of independent Poisson random variables is Poisson-distributed.

From Poisson distribution probability mass function it follows:

$$\begin{aligned} P(K_i = 0) &= \exp(\lambda(l-k+1)) \\ \lambda &= \ln\left(P(K_i = 0)\right)/(l-k+1). \end{aligned}$$

One can estimate the probability not to sequence a particular unique k-mer, $p := P(K_i = 0)$, by the ratio $1 - o/n$. Therefore one can also estimate λ by

$$\hat{\lambda} = \ln\left(1 - o/n\right)/(l-k+1).$$

Next one can investigate the statistical properties of an indicator variable I_i defined as

$$I_i = \begin{cases} 1, & K_i = 0 \ (k\text{-mer in the position } i \text{ has not been seen in the sample}) \\ 0, & K_i > 0 \ (k\text{-mer in the position } i \text{ has been seen in the sample}) \end{cases}$$

The distribution of I_i is

x	1	0
$P(I_i = x)$	p	$(1-p)$

and $\mathrm{E}(I_i) = p$; $\mathrm{Var}(I_i) = p(1-p)$.

Because the starting positions of reads are independent of each other

$$\begin{aligned} \mathrm{E}(I_i \cdot I_{i-1}) =& P(S_i = 0)P(S_{i-1} = 0) \cdot \ldots \cdot P(S_{i-l+k-1} = 0) \\ =& \exp(-\lambda(l-k+2)) \end{aligned}$$

and similarly one can derive a more general result:

$$\mathrm{E}(I_i \cdot I_j) = \begin{cases} \exp(-\lambda(l-k+1+|j-i|)), & \text{if } |j-i| \leq l-k \\ \exp(-2\lambda(l-k+1)), & \text{if } |j-i| > l-k. \end{cases}$$

Therefore one can calculate the covariance between I_i and I_j as

$$\begin{aligned} \mathrm{cov}(I_i, I_j) &= \mathrm{E}(I_i \cdot I_j) - \mathrm{E}(I_i)\mathrm{E}(I_j) \\ &= \begin{cases} \exp(-\lambda(l-k+1+|j-i|)) \\ \quad - \exp(-2\lambda(l-k+1)), & \text{if } |j-i| \leq l-k \\ 0, & \text{if } |j-i| > l-k. \end{cases} \end{aligned}$$

From the derived result one can notice all the covariances to be non-negative (and the covariance decreases as the distance between the i and j increases).

Denote by Ψ the starting positions of all the unique k-mers searched. The sum of I_i's,

$$\sum_{i \in \Psi} I_i,$$

gives the number of unique k-mers not found from the reads. The variance of the sum can be calculated as

$$\mathrm{Var}\left(\sum I_i\right) = n \cdot \mathrm{Var}(I_i) + \sum_{i,j \in \Psi; i \neq j} \mathrm{cov}(I_i, I_j). \tag{2.4}$$

If all (n) unique k-mers are positioned in the genome next to each other, starting from a position i_0, the equation (2.4) takes the form

$$\begin{aligned} \mathrm{Var}\left(\sum I_i\right) = & \sum_{j=0}^{\min(l-k,n-1)} \mathrm{cov}\left(I_{i_0}, I_{i_0+j}\right) \\ & + \sum_{j=-1}^{\min(l-k,n-2)} \mathrm{cov}\left(I_{i_0+1}, I_{i_0+1+j}\right) + \ldots \\ & + \sum_{j=\max(-(l-k),-(z-1))}^{\min(l-k,n-z)} \mathrm{cov}\left(I_{i_0+z}, I_{i_0+z+j}\right) + \ldots \\ & + \sum_{j=\max(-(l-k),-(n-1))}^{0} \mathrm{cov}\left(I_{i_0+n-1}, I_{i_0+n-1+j}\right), \end{aligned}$$

because $\mathrm{cov}(I_i, I_j) = 0 if |j-i| > l-k$.

After rearranging the terms and taking into account that covariance between I_i and I_j depends only from the distance $|j - i|$, and not from the actual position in the genome, eg $\operatorname{cov}(I_i, I_j) = \operatorname{cov}(I_{i+x}, I_{j+x})$, one can write:

$$\begin{aligned}
\operatorname{Var}\left(\sum I_i\right) =& n\operatorname{cov}(I_{i_0}, I_{i_0}) + 2(n-1)\operatorname{cov}(I_{i_0}, I_{i_0+1}) \\
& + 2(n-2)\operatorname{cov}(I_{i_0}, I_{i_0+2}) + \dots \\
& + 2(n - \min(l-k, n-1))\operatorname{cov}\left(I_{i_0}, I_{i_0+\min(l-k,n-1)}\right) \\
=& n\operatorname{Var}(I_{i_0}) + \sum_{j=1}^{\min(l-k,n-1)} 2(n-j)\operatorname{cov}(I_{i_0}, I_{i_0+j}) \\
=& np(1-p) + \sum_{j=1}^{\min(l-k,n-1)} 2(n-j)\left\{\exp(-\lambda(l-k+1+j))\right. \\
& \left. - \exp(-2\lambda(l-k+1))\right\} \qquad (2.5)
\end{aligned}$$

Remark: because the non-negative covariances between I_i and I_j decrease as the distance between the positions i and j increases the formula derived represents the worst case scenario — if the unique k-mers are positioned further away (and not next to each other) then the variance of the sum is smaller than the one given in 2.5.

Because n is constant $\operatorname{Var}(O) = \operatorname{Var}(n - O) = \operatorname{Var}(\sum I_i)$. To get an estimate for $\operatorname{Var}(O)$ one has to plug in the estimates of λ and p to the formula (2.5):

$$\begin{aligned}
\widehat{\operatorname{Var}(O)} &= \widehat{\operatorname{Var}(\sum I_i)} \\
&= n\hat{p}(1-\hat{p}) + \sum_{j=1}^{\min(l-k,n-1)} 2(n-j)\left\{\exp(-\hat{\lambda}(l-k+1+j))\right. \\
&\qquad \left. - \exp(-2\hat{\lambda}(l-k+1))\right\} \\
&= o(1 - o/n) + \sum_{j=1}^{\min(l-k,n-1)} 2(n-j)\left\{\exp(-\hat{\lambda}(l-k+1+j))\right. \\
&\qquad \left. - \exp(-2\hat{\lambda}(l-k+1))\right\}
\end{aligned}$$

The formula acquired is presented as the formula (2.2) in the Formulas section.

Next one can calculate the variance of expected number of unique k-mers (expected for an internal node based on the proportions of species-specific k-mers discovered; denoted by $\hat{E}$). Let $\hat{P}_1$ be the estimator for probability to discover a k-mer specific to species 1 ($\hat{P}_1 = O_1/n_1$) and $\hat{P}_2$ is the estimator for probability to discover a k-mer specific to species 2 ($\hat{P}_2 = O_2/n_2$). Because $\hat{P}_1$ and $\hat{P}_2$ can be considered to be independent (these estimates are likely to be based on different genomes or at least different genome regions of one previously unknown species) one can write:

$$\begin{aligned}
\mathrm{Var}(\hat{E}) &= \mathrm{Var}\left(n_{node}(\hat{P}_1 + \hat{P}_2 - \hat{P}_1 \cdot \hat{P}_2)\right) \\
&= n_{node}^2 \mathrm{Var}\left(\hat{P}_1 + \hat{P}_2 - \hat{P}_1 \cdot \hat{P}_2\right), \\
&= n_{node}^2 \left\{\mathrm{Var}(\hat{P}_1) + \mathrm{Var}(\hat{P}_2) + \mathrm{Var}(\hat{P}_1 \cdot \hat{P}_2) - 2\mathrm{cov}(\hat{P}_1 + \hat{P}_2, \hat{P}_1 \cdot \hat{P}_2)\right\}, \\
&= n_{node}^2 \left\{\mathrm{Var}(\hat{P}_1) + \mathrm{Var}(\hat{P}_2) + \mathrm{Var}(\hat{P}_1 \cdot \hat{P}_2) - 2\mathrm{Var}(\hat{P}_1)\mathrm{E}(\hat{P}_2)\right. \\
&\qquad \left. -2\mathrm{Var}(\hat{P}_2)\mathrm{E}(\hat{P}_1)\right\},
\end{aligned}$$

If $X \perp Y$ then $\mathrm{Var}(X \cdot Y) = \mathrm{Var}(X) \cdot \mathrm{Var}(Y) + \mathrm{Var}(X)\left\{\mathrm{E}(Y)\right\}^2 + \mathrm{Var}(Y)\left\{\mathrm{E}(X)\right\}^2$ and therefore

$$\begin{aligned}
\mathrm{Var}(\hat{E}) =& n_{node}^2 \left\{\mathrm{Var}(\hat{P}_1) + \mathrm{Var}(\hat{P}_2) + \mathrm{Var}(\hat{P}_1) \cdot \mathrm{Var}(\hat{P}_2) + \mathrm{Var}(\hat{P}_1)\mathrm{E}(\hat{P}_2)^2\right. \\
&\quad \left. + \mathrm{Var}(\hat{P}_2)\mathrm{E}(\hat{P}_1)^2 - 2\mathrm{Var}(\hat{P}_1)\mathrm{E}(\hat{P}_2) - 2\mathrm{Var}(\hat{P}_2)\mathrm{E}(\hat{P}_1)\right\} \\
=& n_{node}^2 \left\{\mathrm{Var}(\hat{P}_1)[1 + \mathrm{Var}(\hat{P}_2) + \mathrm{E}(\hat{P}_2)^2 - 2\mathrm{E}(\hat{P}_2)] + \mathrm{Var}(\hat{P}_2)[1 + \mathrm{E}(\hat{P}_1)^2 - 2\mathrm{E}(\hat{P}_1)]\right\}.
\end{aligned}$$

To get an estimate of $\mathrm{Var}(\hat{E})$ one can first notice

$$\begin{aligned}
\mathrm{Var}(\hat{P}_1) &= \mathrm{Var}(O_1/n_1) \\
&= \mathrm{Var}(O_1)/n_1^2 \\
\widehat{\mathrm{Var}(\hat{P}_1)} &= \widehat{\mathrm{Var}(O_1)}/n_1^2,
\end{aligned}$$

where $\widehat{\mathrm{Var}(O_1)}$ can be calculated by using formula (2.2). Similarly one can estimate $\mathrm{Var}(\hat{P}_1)$; $\mathrm{E}(\hat{P}_1)$ can be estimated by $\hat{p}_1$; etc.

After plugging in all the estimates one can write:

$$\begin{aligned}
\widehat{\text{Var}(\hat{E})} =& n_{node}^2 \left(\widehat{\text{Var}(\hat{P}_1)}[1 + \widehat{\text{Var}(\hat{P}_2)} + \hat{p}_2^2 - 2\hat{p}_2] \right. \\
& \left. + \widehat{\text{Var}(\hat{P}_2)}[1 + \hat{p}_1^2 - 2\hat{p}_1] \right) \\
=& n_{node}^2 \left(\frac{\widehat{\text{Var}(O_1)}}{n_1^2}[1 + \widehat{\text{Var}(O_2)}/n_2^2 + \hat{p}_2^2 - 2\hat{p}_2] \right. \\
& \left. + \frac{\widehat{\text{Var}(O_2)}}{n_2^2}[1 + \hat{p}_1^2 - 2\hat{p}_1] \right).
\end{aligned}$$

This completes the derivation of formula (2.3).

Chapter 3

Confidence interval for $\mathrm{E}(O)/e$ ratio

3.1 Formulas

The estimated variance of the logarithm of observed-expected ratio is

$$\widehat{\mathrm{Var}}(\log(O/\hat{E})) = \widehat{\mathrm{Var}(O)}/o^2 + \widehat{\mathrm{Var}(\hat{E})}/\hat{e}^2.$$

Approximate distribution of the logarithm of $O/\hat{E}$-ratio (under some mild assumptions) is

$$\log(O/\hat{E}) \overset{asympt.}{\sim} N\left(\log(\mathrm{E}(O)) - \log(e), \widehat{\mathrm{Var}}(\log(O/\hat{E}))\right),$$

The approximate $(1-\alpha)$-confidence intervals for $\mathrm{E}(O)/e$-ratio

$$\exp\left(\log(o/\hat{e}) + z_{\alpha/2}\sqrt{\widehat{\mathrm{Var}}(\log(O/\hat{E}))}\right)\ldots\exp\left(\log(o/\hat{e}) + z_{1-\alpha/2}\sqrt{\widehat{\mathrm{Var}}(\log(O/\hat{E}))}\right).$$

To calculate the p-value to test $H_0 : \mathrm{E}(O)/e = \theta_0$ one can use the (asymptotically valid) formula:

$$p - value = \Phi\left(-\left|\frac{\ln(o/\hat{e}) - \ln(\theta_0)}{\sqrt{\widehat{\mathrm{Var}}(\log(O/\hat{E}))}}\right|\right)\cdot 2.$$

3.2 Derivation

First one can notice that the proportion of specific k-mers discovered is asymptotically normally distributed if $n \to \infty$ and $0 < p < 1$ (so that both

$np \to \infty$ and $(1-p)n \to \infty$):

$$\sqrt{n}(O/n - p) \xrightarrow{D} N(0; \mathrm{Var}(O)/n),$$

For observed k-mer count of a node therefore approximately holds (if n_{node} is big and $p_{node} > 0$ — due to sequencing errors this is always true — and $p_{node} < 1$, eg the sequencing coverage should not be extremely big):

$$O_{node}/n_{node} \overset{approx.}{\sim} N(p_{node}; \mathrm{Var}(O_{node})/n_{node}^2)$$
$$O_{node} \overset{approx.}{\sim} N(p_{node} n_{node} (= \mathrm{E}(O_{node})); \mathrm{Var}(O_{node})).$$

Reasoning: If unique k-mers are located next to each other then indicator variables showing a particular k-mer is discovered form a strictly stationary stochastic process with strong mixing (α-mixing) property. Under weak assumptions like coverage is greater than zero and less than infinity ($\lambda > 0; \lambda < \infty$) one can show the central limit theorem to hold.

Similarly if $n_{node} \to \infty$ and $0 << p_1, p_2 << 1$, one can show

$$\hat{E}/n_{node} \overset{approx.}{\sim} N(e/n_{node}; D(\hat{E})/n_{node}^2)$$
$$\hat{E} \overset{approx.}{\sim} N(e; \mathrm{Var}(\hat{E})).$$

One can prove the claim above by using the result by Aroian (1947). Aroian showed the product of two independent normal variables $X_1 \sim N(\mu_1, \sigma_1^2)$ and $X_2 \sim N(\mu_2, \sigma_2^2)$ to be approximately normal if $\mu_1/\sigma_1 \to \infty$ and $\mu_2/\sigma_2 \to \infty$; therefore $\hat{E} = \left(\hat{P}_1 + \hat{P}_2 + \hat{P}_1 \cdot \hat{P}_2\right) n_{node}$ can be viewed as a linear combination of a normally distributed variables, if both n_1 and n_2 are big and $0 < p_1 < 1, 0 < p_2 < 1$.

Next one can use delta method to derive the distribution of $\ln(\hat{E})$ and $\ln(O_{node})$. Here are presented the Delta method's main idea (replicated from wikipedia, [2]) :

If the distribution of a statistic X_n converges to normality as the sample size increases,

$$\sqrt{n}(X_n - \theta) \xrightarrow{D} N(0, \sigma^2),$$

where θ and σ^2 are finite valued constants and $\xrightarrow{D}$ denotes convergence in distribution, then

$$\sqrt{n}[g(X_n) - g(\theta)] \xrightarrow{D} \mathcal{N}(0, \sigma^2[g'(\theta)]^2) \tag{3.1}$$

for any function g satisfying the property that $g'(\theta)$ exists and is non-zero valued.

Therefore, by using delta method:

$$\ln(\hat{E}) \sim N(\ln(E); \mathrm{Var}(\hat{E})/e^2)$$

$$\ln(O_{node}) \sim N(\ln(\mathrm{E}(O_{node})); \mathrm{Var}(O_{node})/\mathrm{E}(O_{node})^2).$$

The assumptions of the delta method are only fulfilled if $e > 0$ and $\mathrm{E}(O_{node}) > 0$, but both of these requirements are likely to be satisfied, at least due to sequencing errors.

Because $\hat{E}$ and O_{node} are likely to be independent — probably the k-mers are situated more than a read's length apart — one can write

$$\ln(O_{node}) - \ln(\hat{E}) \sim N(\ln(\mathrm{E}(O_{node}) - \ln(e); \mathrm{Var}(O_{node})/\mathrm{E}(O_{node})^2 + \mathrm{Var}(\hat{E})/e^2)$$
$$\ln(O_{node}/\hat{E}) \sim N(\ln(\mathrm{E}(O_{node}/e)); \mathrm{Var}(O_{node})/\mathrm{E}(O_{node})^2 + \mathrm{Var}(\hat{E})/e^2).$$

One can conclude from the formula above that an estimator for the variance of logarithm of observed-expected ratio is:

$$\widehat{\mathrm{Var}}\left(\ln(O_{node}/\hat{E})\right) = \widehat{\mathrm{Var}}(O_{node})/o_{node}^2 + \widehat{\mathrm{Var}}(\hat{E})/\hat{e}^2.$$

Now one can derive a test procedure to test hypothesis about the ratio $\mathrm{E}(O_{node}/e)$. One can use the z-test to test the hypothesis $H_0 : \mathrm{E}(O_{node}/e) = \theta_0$:

$$\frac{\ln(O_{node}/\hat{E}) - \ln(\theta_0)}{\sqrt{\widehat{\mathrm{Var}}\left(\ln(O_{node}/\hat{E})\right)}} \overset{H_0,approx.}{\sim} N(0,1)$$

and the p-value of the two-sided test can be calculated as

$$p - value = \Phi\left(-\left|\frac{\ln(O_{node}/\hat{E}) - \ln(\theta_0)}{\sqrt{\widehat{\mathrm{Var}}\left(\ln(O_{node}/\hat{E})\right)}}\right|\right) \cdot 2.$$

Also one can get the approximate $1-\alpha$-confidence interval for $\ln(\mathrm{E}(O_{node}/e))$ as:

$$\ln(O_{node}/\hat{e}) \pm z_{\alpha/2} \cdot \widehat{\mathrm{Var}}(\ln(O_{node}/\hat{E})).$$

Bibliography

[1] Illumina. *Estimating Sequencing Coverage* (2001); http://res.illumina.com/documents/products/technotes/technote_coverage_calculation.pdf (accessed April 16, 2015).

[2] Wikipedia contributors, "Delta method," *Wikipedia, The Free Encyclopedia*, http://en.wikipedia.org/w/index.php?title=Delta_method&oldid=656023015 (accessed April 16, 2015).
